# Mediated roles of generalized trust and perceived social support in the effects of problematic social media use on mental health: A cross‐sectional study

**DOI:** 10.1111/hex.13169

**Published:** 2020-11-30

**Authors:** Chung‐Ying Lin, Peyman Namdar, Mark D. Griffiths, Amir H. Pakpour

**Affiliations:** ^1^ Institute of Allied Health Sciences, National Cheng Kung University Hospital, College of Medicine National Cheng Kung University Tainan Taiwan; ^2^ Social Determinants of Health Research Center Research Institute for Prevention of Non‐Communicable Diseases Qazvin University of Medical Sciences Qazvin Iran; ^3^ International Gaming Research Unit Psychology Department Nottingham Trent University Nottingham UK; ^4^ Department of Nursing School of Health and Welfare Jönköping University Jönköping Sweden

**Keywords:** anxiety, depression, happy, mental quality of life, problematic social media use

## Abstract

**Background:**

Current literature lacks evidence concerning how problematic social media use associates with mental health. To address the gap, the present study used mediation models to examine whether generalized trust and perceived social support (PSS) are potential mediators in the relationship between problematic social media use and mental health.

**Methods:**

The sample comprised Iranian adults (n = 1073; 614 females). The participants completed a number of scales to assess problematic social media use (Bergen Social Media Addiction Scale), generalized trust (Generalized Trust Scale), PSS (Multidimensional Scale of Perceived Social Support, happiness (Oxford Happiness Questionnaire Short Form), depression and anxiety (Hospital Anxiety and Depression Scale), and mental quality of life (Short Form‐12).

**Results:**

Problematic social media use had negative effects on happiness and mental quality of life via the mediators of generalized trust (bootstrapping SE = 0.017; effect = −0.041; 95% CI = −0.079, −0.012) and PSS (bootstrapping SE = 0.023; effect = −0.163; 95% CI = −0.211, −0.119). Problematic social media use had positive effects on anxiety and depression via the mediators of generalized trust (bootstrapping SE = 0.022; effect = 0.064; 95% CI = 0.026, 0.113) and PSS (bootstrapping SE = 0.024; effect = 0.052; 95% CI = 0.009, 0.102).

**Conclusions:**

Problematic social media use, generalized trust and PSS are important factors for an individual's mental health. Health‐care providers may want to assist individuals regardless of having mental health problems in reducing their problematic social media use and improving their generalized trust and social support.

## 1 INTRODUCTION

The World Health Organization^1^ has defined mental health as the ‘subjective well‐being, perceived self‐efficacy, autonomy, competence, intergenerational dependence, and self‐actualization of one's intellectual and emotional potential, among others…It is, however, generally agreed that mental health is broader than a lack of mental disorders’ (p. 5). Therefore, mental health encompasses a broad spectrum with both positive and negative outcomes. From a positive perspective, mental health includes happiness and a good mental quality of life. From a negative perspective, good mental health includes the absence of disorders such as anxiety and depression.^2^ Literature further shows and suggests that mental health can be a basis and a reciprocal consequence of an individual's overall health, including physical wellness and social burden.^2^, ^3^ Indeed, a large‐scale study conducted in Taiwan showed that mental health problems (ie psychological symptoms) might lead to lowered quality of life in various domains (social, physical and environmental) among 1080 healthy workers.^4^ Therefore, improving mental health has been proposed to be an important topic for public health.^2^, ^3^

Before health‐care providers can design effective programmes to improve mental health among general populations, they need to identify relevant factors that are related to good and/or poor mental health. Previous empirical studies have demonstrated that poor mental health is related to lower generalized trust,^5^, ^6^ lower perceived social support (PSS)^7^ and problematic social media use.^8^ Consequently, higher levels of both generalized trust and PSS are related to better mental health (including increased happiness, elevated mental quality of life, reduced anxiety and decreased depression). On the other hand, problematic social media use is related to poorer mental health.

Generalized trust (aka *general trust* or *trust*) is defined as ‘a willingness to be vulnerable to the actions of others’ (p. 1)^9^ and is the basis of societal factors that help maintain daily living of an individual. Also, generalized trust supplements PSS in contributing to good social relationships. Indeed, evidence shows that trust makes an individual anticipate positive rather than negative outcomes from others’ actions.^10^, ^11^, ^12^ Consequently, a positive relationship is then established based on the positive interaction between two individuals. A positive relationship may further assist an individual in obtaining health benefits, including good psychological health, low levels of distress and happy mood.^13^, ^14^, ^15^, ^16^, ^17^

Perceived social support is defined as ‘perceptions of the extent to which individuals from one's social network are available to provide social support’ (p. 85)^7^ and is the basis of a good social relationship. A greater level of PSS often leads to satisfactory social interaction and consequently results in improved mental health and the avoidance of psychological distress.^18^, ^19^ Moreover, PSS has been reported as having larger effects concerning mental health than actual received social support.^20^ Therefore, assessing how an individual perceives social support may provide some insights for health‐care providers to an individual's mental health.

Problematic social media use, a term that is sometimes interchangeably used with *social media addiction* or *social media overuse*, currently does not have a consensus in its definition among researchers.^21^ Although there is no clear definition of problematic social media use, current literature has demonstrated consistent findings in the relationship between problematic social media use (or other related terms) and mental health. More specifically, problematic social media use has been related to psychological distress, including stress, anxiety and depression.^8^, ^22^, ^23^ Individuals with problematic social media use may suffer from *fear of missing out* which may result in mental health problems for some individuals.^24^ Apart from the relationships with mental health problems, the literature reports that problematic social media is also related to poor trust and low levels of PSS.^25^, ^26^

Problematic social media use has become a public issue worldwide over the past decade for a number of reasons. First, the growth of global social media use has been rapid given the advances in technology on the internet and smartphone.^27^, ^28^ Data from 2020 reported that 3.6 billion people worldwide who had accessed the internet were social media users.^29^ More specifically, many social media platforms (eg Twitter, Instagram, Facebook) have been developed with the advances of internet/smartphone technology. These very popular and attractive sites are designed to keep users engaged and for many individuals their use can be habitual and/or excessive taking time away from activities that may be more important such as educational and/or occupational duties. Consequently, excessive social media use, like other types of excessive use such as excessive internet or smartphone use, may cause a variety of health problems, including sleep problems, musculoskeletal discomfort, mood issues and impaired daily functions, as well as addiction‐like consequences in a small minority of individuals.^27^, ^28^, ^29^, ^30^, ^31^, ^32^, ^33^, ^34^, ^35^, ^36^

Empirical evidence also shows that poor mental health is related to low levels of generalized trust and PSS, as well as problematic social media use.^7^, ^13^, ^22^ However, previous research has rarely examined simultaneously whether generalized trust, PSS, and problematic social media use are all directly related to mental health. To address the literature gap regarding direct or indirect relationships, the present study used a phased cross‐sectional design to examine whether generalized trust and PSS were mediators in the relationship between problematic social media use and different types of mental health (including happiness, depression, anxiety and mental quality of life). The present authors hypothesized that generalized trust and PSS would be mediators because prior research has shown that problematic social media may lead to reduced trust and lowered PSS.^25^, ^26^

## 2 METHODS

### 2.1 Participants and procedure

The study sample was recruited from health centres and health houses in Qazvin province, Iran. A two‐level cluster sampling design was utilized. More specifically, (a) three health centres and two health houses were randomly selected in each city (Qazvin, Takestan, Abyek and Buin Zahra) in Qazvin province and (b) using a convenience sampling approach, participants were selected from those individuals who had been referred to health centres and health houses to receive primary health care. To be eligible, participants had to be adult (ie 18 years of age or older) and agree to participate. Qazvin University of Medical Sciences’ ethics committee approved the study, and all study participants gave their written informed consent (IR.QUMS.REC.1397.122).

After participants had provided informed consent, they completed a baseline questionnaire of problematic social media use. The same participants completed the first follow‐up measures on PSS and social trust one month after the baseline assessment. Finally, the participants completed a measure relating to depression, anxiety, quality of life and happiness three months after the baseline assessment. All the assessments were completed between May and November 2019.

### 2.2 Measures

#### 2.2.1 Bergen Social Media Addiction Scale (BSMAS)

The BSMAS,^22^ including the Persian version,^8^ comprises six items rated on a five‐point Likert scale to assess problematic social media use. The six items utilize the addiction components model's six core criteria.^37^, ^38^ A sample item for the BSMAS is ‘How often during the last week have you spent a lot of time thinking about social media or planned use of social media?’ A higher score in the BSMAS indicates an individual is more at risk of developing problematic social media use. The BSMAS has previously been translated into Persian for use among Iranians and underwent a rigorous procedure to ensure its linguistic validity. The Cronbach's alpha in the present study was 0.86.

#### 2.2.2 Generalized Trust Scale (GTS)

The GTS comprises six items rated on a five‐point Likert scale to assess generalized social trust.^12^, ^39^ A sample item of the GTS is ‘Most people are basically honest’. A higher score on the GTS indicates a greater level of generalized trust. Because there was no previous Persian validation of GTS, the GTS was translated into Persian following international guidelines.^40^, ^41^ The Cronbach's alpha in the present study was 0.89.

#### 2.2.3 Multidimensional Scale of Perceived Social Support (MSPSS)

The MSPSS,^42^ including the Persian version,^43^ comprises 12 items rated on a seven‐point Likert scale to assess PSS comprising three dimensions, including four items related to family, four items related to friends, and four items related to significant others. A sample item of the MSPSS family dimension is ‘My family really tries to help me’; a sample item of the MSPSS friends dimension is ‘My friends really try to help me’; and a sample item of the MSPSS friends dimension is ‘There is a special person who is around when I am in need.’ Higher scores on the MSPSS indicate a stronger level of PSS. The Cronbach's alpha in the present study was 0.82.

#### 2.2.4 Oxford Happiness Questionnaire Short Form (OHQ‐SF)

The OHQ‐SF,^44^ including the Persian version,^45^ comprises eight items rated on a six‐point Likert scale to assess happiness. A sample item of the OHQ‐SF is ‘I feel that life is very rewarding’. Higher scores on the OHQ‐SF indicate a higher level of happiness. The Cronbach's alpha in the present study was 0.87.

#### 2.2.5 Hospital Anxiety and Depression Scale (HADS)

The HADS, including the Persian version,^46^, ^47^ comprises 14 items rated on a four‐point Likert scale that assess depression, anxiety, and psychological distress. The 14 items are equally distributed on a seven‐item depression subscale and a seven‐item anxiety subscale. A sample item of HADS anxiety is ‘I feel tense or wound up’ and for HADS depression is ‘I feel as if I am slowed down’. Higher scores on the HADS indicate a more severe level of depression or anxiety. The Cronbach's alpha in the present study was 0.80 and 0.83 for anxiety and depression subscales, respectively.

#### 2.2.6 Short Form‐12 (SF‐12)

The SF‐12,^48^ including the Persian version,^49^ comprises 12 items rated on either a Likert scale from 3 to 6 points or a dichotomous scale (yes and no) and assesses quality of life, including mental and physical quality of life. A sample item of the SF‐12 physical component score is ‘Does your health now limit you from climbing several flights of stairs?’ and a sample item of the SF‐12 mental component score is ‘Have you felt downhearted and blue?’ Higher scores on the SF‐12 indicate a better quality of life. In the present study, only mental quality of life component score of the SF‐12 was used for analysis. The Cronbach's alpha in the present study was 0.79.

### 2.3 Statistical analysis

**Figure 1:**
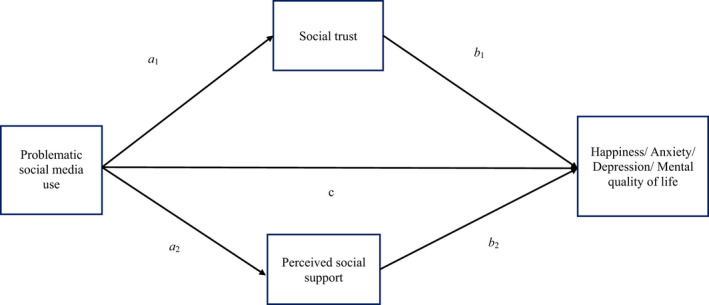
An illustration of proposed mediation model. Generalized trust and perceived social support were proposed mediators of the effect of problematic social media use on people's mental health (including happiness, anxiety, depression and mental quality of life). Different types of mental health were analysed in separate mediation models. **P < .001. ^†^Age, sex and place of residence were adjusted for the model

After using descriptive statistics to examine the sociodemographic features of the participants, Pearson correlations were applied to understand how the studied variables (ie problematic social media use, generalized trust, PSS, happiness, anxiety, depression and mental quality of life) were related. Following this, four mediation models were constructed and all shared the same controlled variables (ie age, gender, marital status, having children or not, place of resident, occupational status and years of education), mediators (ie PSS and generalized trust), and independent variable (ie problematic social media use). The differences among the four mediation models were the use of different dependent variables, that is, each mediation model had a different type of mental health (ie happiness, anxiety, depression and mental quality of life; see Figure 1). With the use of 5000 bootstrapping resamples, all the mediation models were assessed using Model 4 in PROCESS macro for SPSS.^50^

The PROCESS macro based on the linear regression models was used to construct mediation models with the use of the bootstrapping method. More specifically, the associations between variables (ie the associations between independent variables and mediators; between mediators and dependent variables; and between independent variables and dependent variables) were estimated using the ordinary least square method. The significance of the direct effects was examined using independent *t* tests; the significance of the indirect effects was examined using the bootstrapping method (ie if the 95% samples of the bootstrapping resamples do not cover 0, the mediating effect is significant). Moreover, all the independent variables, mediators and dependent variables were treated as numeric variables in the mediation models. This treatment is appropriate given that all the independent variables, mediators, and dependent variables were summed total scores of validated psychometric scales.

## 3 RESULTS

The participants’ mean age was 36.57 years (SD = 10.21), and their mean number of years spent in education was 9.87 years (SD = 4.68). More than half of the participants were females (n = 614, 57.2%), had children (n = 663, 61.8%) and lived in urban areas (n = 703, 65.5%). Nearly half of the participants were married (n = 571, 49.3%) and employed (n = 524, 48.8%; Table 1).

**Table 1:** Sociodemographic characteristics of participants (n = 1073)

	Mean or n	SD or %
Age (y)	36.57	10.21
Educational year	9.87	4.68
Gender		
Male	459	42.8%
Female	614	57.2%
Marital status		
Single	311	30.7%
Married	571	49.3%
Divorced	191	20.0%
Having children		
Yes	663	61.8%
No	410	38.2%
Place of residence		
Urban	703	65.5%
Rural	370	34.5%
Occupational status		
Employed	524	48.8%
Unemployed	179	16.7%
Student	85	7.9%
Housewife	232	21.6%
Retired	53	4.9%

Problematic social media use (mean = 14.28, SD = 4.19), generalized trust (mean = 3.02, SD = 1.00), PSS (mean = 63.57, SD = 10.34), happiness (Mean = 25.41, SD = 4.39), anxiety (mean = 8.28, SD = 4.16), depression (mean = 7.59, SD = 3.88) and mental quality of life (mean = 50.39, SD = 10.62) were all normally distributed with skewness between −0.60 and 1.81; kurtosis between −0.68 and 1.67 (Table 2) additionally reports that the bivariate correlations between the studied variables were all significant (*P* < .01). More specifically, problematic social media use was negatively related to generalized trust (*r* = −.407), PSS (*r* = −.244), happiness (*r* = −.323) and mental quality of life (*r* = .128), and positively related to depression (*r* = .195) and anxiety (*r* = .170). Generalized trust was negatively related to depression (*r* = −.249) and anxiety (*r* = −.223); and positively related to mental quality of life (*r* = .307). PSS was negatively related to depression (*r* = −.518) and anxiety (*r* = −.335), and positively related to mental quality of life (*r* = .404).

**Table 2:** Pearson correlation matrix of the variables of interest

	*r*							Mean	SD	Skewness	Kurtosis	α
	1	2	3	4	5	6	7					
1. Problematic social media use^a^	1	−0.407	−0.244	−0.323	0.170	0.195	−0.128	14.28	4.19	0.99	0.81	0.82
2. Generalized trust^b^		1	0.259	0.565	−0.223	−0.249	0.307	3.02	1.00	−0.48	−0.14	0.88
3. Perceived social support^c^			1	0.352	−0.335	−0.518	0.404	63.57	10.34	−0.60	−0.68	0.72
4. Happiness^d^				1	−0.139	−0.233	0.230	25.41	4.39	1.31	1.67	0.80
5. Anxiety^e^					1	0.572	−0.289	8.28	4.16	1.81	1.60	0.90
6. Depression^e^						1	−0.301	7.59	3.88	−0.41	−0.42	0.88
7. Mental quality of life^f^							1	50.39	10.62	−0.24	−0.46	0.86

Note

All *P*‐values < .01.

^a^ Assessed using the Bergen Social Media Addiction Scale (BSMAS).

^b^ Assessed using the Generalized Trust Scale (GTS).

^c^ Assessed using the Multidimensional Scale of Perceived Social Support (MSPSS).

^d^ Assessed using the Oxford Happiness Questionnaire Short Form (OHQ‐SF).

^e^ Assessed using the Hospital Anxiety and Depression Scale (HADS).

^f^ Assessed using the Mental Component Summary in the Short Form‐12 (SF‐12).

The mediation models (Table 3) showed that problematic social media use had negative effects on happiness directly (*P* = .016; coefficient = −.090; SE = 0.037) and indirectly via generalized trust (bootstrapping SE = 0.017; effect = −0.041; 95% CI = −0.079, −0.012) and PSS (bootstrapping SE = 0.023; effect = −0.163; 95% CI = −0.211, −0.119). Problematic social media use had positive effects on anxiety indirectly via generalized trust (bootstrapping SE = 0.022; effect = 0.064; 95% CI = 0.026, 0.113) and PSS (bootstrapping SE = 0.019; effect = 0.047; 95% CI = 0.009, 0.085) but not directly (*P* = .348; coefficient = 0.045; SE = 0.047). Problematic social media use had positive effects on depression indirectly via generalized trust (bootstrapping SE = 0.035; effect = 0.140; 95% CI = 0.075, 0.212) and PSS (bootstrapping SE = 0.024; effect = 0.052; 95% CI = 0.009, 0.102) but not directly (*P* = .511; coefficient = 0.037; SE = 0.057). Problematic social media use had negative effects on mental quality of life indirectly via generalized trust (bootstrapping SE = 0.063; effect = −0.181; 95% CI = −0.316, −0.072) and PSS (bootstrapping SE = 0.044; effect = −0.193; 95% CI = −0.286, −0.110) but not directly (*P* = .363; coefficient = 0.096; SE = 0.105).

**Table 3:** Problematic social media use effect on happiness, anxiety, depression, and mental quality of life with mediators of perceived social support and generalized trust

	Coefficient	SE	*t‐*value	*P*‐value
(A) Test on happiness				
Total effect of problematic social media use on happiness	−0.294	0.039	−7.45	<.001
Direct effect of problematic social media use on happiness in mediated model	−0.090	0.037	−2.42	.016
Indirect effect of problematic social media use on happiness	Effect	Boot SE	Boot LLCI	Boot ULCI
Total indirect effect	−0.205	0.028	−0.262	−.153
Indirect effect via generalized social trust	−0.041	0.017	−0.079	−.012
Indirect effect via perceived social support	−0.163	0.023	−0.211	−.119
(B) Test on anxiety				
Total effect of problematic social media use on anxiety	0.156	0.045	3.464	.001
Direct effect of problematic social media use on anxiety in mediated model	0.045	0.047	0.940	.348
Indirect effect of problematic social media use on anxiety	Effect	Boot SE	Boot LLCI	Boot ULCI
Total indirect effect	0.111	0.028	0.058	.170
Indirect effect via generalized social trust	0.064	0.022	0.026	.113
Indirect effect via perceived social support	0.047	0.019	0.009	.085
(C) Test on depression				
Total effect of problematic social media use on depression	0.229	0.059	3.861	.001
Direct effect of problematic social media use on depression in mediated model	0.037	0.057	0.657	.511
Indirect effect of problematic social media use on depression	Effect	Boot SE	Boot LLCI	Boot ULCI
Total indirect effect	0.191	0.041	0.113	.277
Indirect effect via generalized social trust	0.140	0.035	0.075	.212
Indirect effect via perceived social support	0.052	0.024	0.009	.102
(D) Test on mental quality of life				
Total effect of problematic social media use on mental quality of life	−0.279	0.105	−2.659	.008
Direct effect of problematic social media use on mental quality of life in mediated model	0.096	0.105	0.910	.363
Indirect effect of problematic social media use on mental quality of life	Effect	Boot SE	Boot LLCI	Boot ULCI
Total indirect effect	−0.375	0.074	−0.527	−.234
Indirect effect via generalized social trust	−0.181	0.063	−0.316	−.072
Indirect effect via perceived social support	−0.193	0.044	−0.286	−.110

Note

Age, gender, marital status, having children, place of resident, occupational status, and years of education were adjusted for all the models.

Boot = Bootstrapping; Boot SE = bootstrapping standard error; CI 95% = 95% confidence interval using 5000 bootstrap samples; LLCI = lower limit confidence interval; ULCI = upper limit confidence interval; SE = standard error.

## 4 DISCUSSION

With the use of Hayes^50^ mediation model that adopts the bootstrapping method, the present study showed that problematic social media use might impact negatively on mental health (including happiness and mental quality of life) and increase psychological distress (including anxiety and depression). Moreover, the problematic social media effects on happiness included direct effects and indirect effects via both generalized trust and PSS. More specifically, for every one‐point score increase by individuals on the BSMAS, the individual directly had a 0.090 lower point score in happiness and indirectly had a 0.041 lower point score in happiness via generalized trust, and a 0.163 lower point score in happiness via PSS. The problematic social media effects on psychological distress and mental quality of life were only indirect effects via generalized trust and PSS. Moreover, for every one‐point score increase by individuals on the BSMAS, the individual indirectly had a 0.064 higher point score in anxiety via generalized trust, and a 0.047 higher point score in anxiety via PSS; indirectly they had a 0.140 higher point score in depression via generalized trust and a 0.052 higher point score in depression via PSS; also indirectly they had a 0.181 lower point score in mental quality of life via generalized trust and a 0.193 lower point score in mental quality of life via PSS.

The present study's findings concur with previous research results that problematic social media use is a significant factor greatly related to poor mental health.^51^, ^52^ Moreover, Wong et al^52^ proposed that problematic social media use may result in functional impairment, negative social comparison, excessive escapism, and poor sleep. Impacts on the aforementioned factors resulting from problematic social media may subsequently cause mental health problems. In addition to Wong et al’s postulation,^52^ Elhai et al^53^ reviewed 23 studies and concluded that another possible psychopathology in the relationship between problematic social media use and psychological distress was the concept of fear of missing out (FoMO). Through this specific fear, individuals’ stress and level of burnout are possibly increased.

The concept of FoMO may also induce problems concerning generalized trust and PSS. Lai et al^54^ investigated how FoMO correlated with social exclusion and inclusion in a neurobiological context. Their findings indicated that individuals with FoMO were more sensitive towards social inclusive experiences as compared to social exclusion. Consequently, individuals with higher FoMO might be vulnerable in social relationships and they have a high need for approval.^55^ With FoMO, individuals with problematic social media use may generate trust problems (ie not trusting others) and PSS issues (ie feeling isolated and alone). Therefore, researchers are encouraged to utilize the findings of the present study to further clarify whether FoMO is an important factor in linking problematic social media use, trust, and PSS. Moreover, health‐care providers may want to design intervention and prevention programmes to minimize problematic social media use among the general population. The present study showed that problematic social media use was associated with different types of negative mental health consequences and poorer psychological well‐being (eg depression, anxiety). Therefore, minimizing problematic social media use among the general population may prevent subsequent mental health problems. For example, health‐care providers may design prevention programs utilizing motivational interviewing techniques and/or mindfulness to help the general population use social media more appropriately.

Similar to previous findings,^13^, ^14^, ^15^, ^16^, ^17^ the present study showed that generalized trust was related to mental health, including elevated happiness, improved mental quality of life, reduced anxiety, and decreased depression. The aforementioned findings are important for researchers and health‐care providers. More specifically, researchers may build on the findings to further investigate the underlying mechanism of why generalized trust contributes to better mental health outcomes. For example, prior research has proposed that positive mental health is associated with generalized trust and is due to the good social relationships induced by the generalized trust.^9^ In terms of health‐care implications, health‐care providers may consider designing programs that can enhance individuals’ generalized trust. If generalized trust can be elevated among individuals, it is likely that the overall mental health at a population level can be improved.

An association between PSS and mental health was clearly observed in the present study. Moreover, such finding is consistent to the literature. More specifically, when individuals perceive a greater level of social support, they often have satisfactory social interaction, good mental health, and little psychological distress.^18^, ^19^ PSS may minimize the feeling of loneliness and improve leisure activity performance (eg having partners to engage in leisure activities that cannot be done alone) for an individual.^19^ Given that loneliness and lack of leisure are risk factors contributing to poor mental health, a high level of PSS is likely to prevent an individual from developing poor mental health.

Regarding the clinical significance of the present study's findings, the guidance proposed by Cohen^56^ was applied: *r* = .1 indicates small effect; .3 indicates medium effect; and .5 indicates large effect. Therefore, the Pearson correlations shown in Table 2 indicate that problematic social media use had a near large effect on generalized trust and small to medium effects on PSS and mental health. Generalized trust had small to large effects on mental health, and PSS had medium to large effects on mental health. Given that most of the effects were medium to large, the clinical significance of problematic social media use, generalized trust, and PSS on individuals’ mental health is supported.

The present study clearly has some limitations. First, the study sample was recruited through convenience sampling from one Iranian city. Therefore, generalizing the study's results is limited given that many factors (eg generalized trust and PSS) assessed in the present study are likely to be influenced by culture. Following this limitation, future studies need to replicate the present study's findings in different countries to examine the robustness of the mediation models reported here. Second, all the measures were of a self‐report nature. Therefore, common problems from self‐reports including social desirability and memory recall might have influenced the present study's findings. However, given that all the measures used in the present study had robust psychometric properties, the problems of recall bias and social desirability may not be as serious. Further studies should utilize more objective measures (eg Hamilton Depression Rating Scale for depression) to corroborate the present study's findings. Finally and importantly, the present study used a cross‐sectional design to examine the mediation models. Given that time is an important concept in testing mediating effects, the present study suffers from the lack of evidence in determining a causal relationship. More specifically, the present findings cannot provide evidence in the time sequence between the tested independent variable (ie problematic social media use), the mediators (ie PSS and generalized trust) and the dependent variables (ie happiness, anxiety, depression and mental quality of life). Therefore, future studies with longitudinal design are needed to further clarify the causal relationships between these aforementioned variables.

## 5 CONCLUSION

Problematic social media use, generalized trust, and PSS are important factors for an individual's mental health. With less problematic social media use, an individual may have better mental health and lower psychological distress. Moreover, with high levels of generalized trust and PSS, an individual may have good mental health. Therefore, health‐care providers may want to assist individuals (irrespective of whether they have mental health problems) in reducing their problematic social media use and improving their generalized trust and social support.

## CONFLICT OF INTEREST

The authors have declared that no competing interests exist.

## Data Availability

There are no data available for sharing because of conditions imposed by the Ethics approval under which this research was conducted.
